# Neoadjuvant Therapy in Patients with Pancreatic Cancer: A Disappointing Therapeutic Approach?

**DOI:** 10.3390/cancers3022286

**Published:** 2011-05-09

**Authors:** Carolin Zimmermann, Gunnar Folprecht, Daniel Zips, Christian Pilarsky, Hans Detlev Saeger, Robert Grutzmann

**Affiliations:** 1 Department for General, Thoracic and Vascular Surgery and University Cancer Center University Hospital “Carl Gustav Carus”, Technical University Dresden, Germany; 2 Medical Department I and University Cancer Center, University Hospital “Carl Gustav Carus”, Technical University Dresden, Germany; 3 Department of Radiation Oncology and University Cancer Center University Hospital “Carl Gustav Carus”, Technical University Dresden, Germany

**Keywords:** pancreas cancer, neoadjuvant therapy, adjuvant, radiotherapy, chemotherapy

## Abstract

Pancreatic cancer is a devastating disease. It is the fourth leading cause of cancer-related death in Germany. The incidence in 2003/2004 was 16 cases per 100.000 inhabitants. Of all carcinomas, pancreatic cancer has the highest mortality rate, with one- and five-year survival rates of 25% and less than 5%, respectively, regardless of the stage at diagnosis. These low survival rates demonstrate the poor prognosis of this carcinoma. Previous therapeutic approaches including surgical resection combined with adjuvant therapy or palliative chemoradiation have not achieved satisfactory results with respect to overall survival. Therefore, it is necessary to evaluate new therapeutic approaches. Neoadjuvant therapy is an interesting therapeutic option for patients with pancreatic cancer. For selected patients with borderline or unresectable disease, neoadjuvant therapy offers the potential for tumor downstaging, increasing the probability of a margin-negative resection and decreasing the occurrence of lymph node metastasis. Currently, there is no universally accepted approach for treating patients with pancreatic cancer in the neoadjuvant setting. In this review, the most common neoadjuvant strategies will be described, compared and discussed.

## 1. Introduction

Pancreatic cancer is a devastating disease. It is the fourth leading cause of cancer-related death in Germany [1]. The incidence in 2003/2004 was 16 cases per 100.000 inhabitants [1]. Of all carcinomas, pancreatic cancer has the highest mortality rate, with one- and five-year survival rates of 25% and less than 5%, respectively [2].

The etiology of pancreatic cancer has not yet been absolutely clarified. Reported risk factors include chronic pancreatitis, depending on the disease duration; nicotine abuse; alcohol use, as risk factor for chronic pancreatitis; exposure to carcinogenic substances such as vinyl chloride and acrylamide; and genetic factors, for example hereditary pancreatitis, a family history of pancreatic cancer and diabetes mellitus and specific syndromes, like FAMM, HNPCC and Peutz-Jeghers syndrome [3].

Pancreatic cancer is a disease with an extremely poor prognosis. Because of the anatomic location of the tumor, patients with pancreatic cancer do not show any specific early clinical signs, and the disease has a propensity for early dissemination to lymph nodes and other organs [2]. Approximately 80% of patients are metastatic when they are diagnosed, with metastases in the following areas in decreasing order of frequency: regional lymph nodes, liver, lung, adrenal gland, kidney, pleura, peritoneum and bone [4].

Diagnosis is performed by ultrasound and color Doppler imaging (CDI), ultrafast helical computed tomography (CT), endosonography (EUS) or magnetic resonance imaging (MRI) [5,6].

The only curative therapy is resection of the tumor. However, today only approximately 20 percent of patients with pancreatic cancer undergo surgery with curative intent. However, the median survival time of patients whose tumors were resected is only 13 to 25 months with a 5-year overall survival of 15% to 20% [7]. Patients with unresectable tumors and distant metastasis are treated palliatively, for example, they get chemotherapy with gemcitabine in combination with erlotinib and platins and radiation [8].

Moreover the above-mentioned facts support the concept that patients with pancreatic cancer suffer from a systemic disease at the time of diagnosis, and this emphasizes the need for a multimodal approach to treatment, including next to surgery chemotherapy, radiation or both together.

During the past several years, these therapeutic options have been used mainly in the adjuvant setting. The use of these therapies in the neoadjuvant setting has been studied and has many advantages.

In this review, the most common neoadjuvant settings in pancreatic cancer will be described, compared and discussed in a retrospective way.

## 2. The Use of Neoadjuvant Therapy

The use of neoadjuvant therapy can be justified by many arguments. First, neoadjuvant therapy can be used to treat all eligible patients. Moreover, multimodal therapy is likely to be better tolerated prior to radical pancreaticoduodenectomy because no recovery time from surgery is necessary in the adjuvant setting in this case. Because of this, more patients will be able to receive full-dose chemotherapy and radiotherapy. Consequently, the use of full doses may possibly enhance the effectiveness of the combined treatment. Neoadjuvant therapy also avoids the delays to systemic and local treatment. For patients with postoperative complications, it is therefore not possible to begin the necessary adjuvant therapy soon after surgery.

If patients are responder, this treatment increases the potentially curative resection rates by reducing the bulk of the primary tumor [9]. Increasing the curative resection rate is urgent due to the current resection rates of only 20–30%. After performing administering neoadjuvant radio-/chemotherapy to patients with unresectable tumors, resection rates of 51% were reported [10]. The downstaging may allow a greater chance of complete resection and higher negative margin rates. Thus, the decrease in the frequency of positive surgical margins allows more cancer patients to achieve oncological R0 resection. A positive margin is one of the most significant prognostic markers for overall and disease-free survival following surgery [11].

Furthermore, neoadjuvant therapy may allow a reduction of lymph node metastasis and vascular involvement, leading to an improvement in prognosis and a reduction of the local recurrence rate. This fact was demonstrated in a retrospective analysis by Greer *et al.* [12]. Data for 102 patients were compared; of the 102 patients, 19 patients were treated only with surgery, 41 patients subsequently received adjuvant therapy, and 42 patients achieved neoadjuvant therapy. In the group of patients treated with neoadjuvants, only one of the 28 patients (4%) experienced a local recurrence. In contrast, in the adjuvant group, four local recurrences in nine patients (44%) were registered [12].

Additionally, neoadjuvant therapy could prevent peritoneal tumor cell implantation and dissemination during surgery [13].

Patients who develop a progressive disease under neoadjuvant treatment will be identified and not selected for radical surgery because probably they will not enjoy a survival benefit. Therefore, these patients are spared unnecessary surgery and the potential complications. For patients with aggressive and rapidly progressing disease, the quality of life is paramount.

Given the tumor biology in untreated tumor tissue, neoadjuvant therapies will be more effective because of the better oxygenation and an intact vascular bed [14]. These may enhance the radiation effect by simultaneous administration of cytostatics.

Another important advantage is the probable improvement of operability after successful neoadjuvant therapy. Increased complication and mortality rates were not demonstrated in previous studies. Lower rates of postoperative fistula and anastomotic leakage of the pancreatojejunostomy after neoadjuvant therapy have been reported [9,15-17]. This is due to the increased fibrosis of the pancreas induced by the combined radio-/chemotherapy. Furthermore, in retrospective and prospective studies, comparable operating times were found after neoadjuvant therapy was performed [9].

There are also many disadvantages that should be discussed. Neoadjuvant therapy can only be applied when the diagnosis is confirmed by histology. This fact requires a fully committed team of multidisciplinary specialists who are able to perform an endosonographically or CT-guided biopsy and laparoscopy; whereas the indication of adjuvant therapy is based on the surgical and pathologic staging.

Neoadjuvant therapy could also be a potential overtreatment for patients who would be cured with curative surgery and who do not respond to neoadjuvant therapy. As of today prediction of the use of neoadjuvant therapy is not possible. However, pancreatic cancer is a systemic disease that should be treated as soon as possible.

## 3. Neoadjuvant Therapy for the Treatment of Resectable Disease

Table 1 lists a summary of the non-randomized neoadjuvant trials that have been conducted since the 1990s. The majority of these studies was conducted in the United States and utilized multimodal regimes (Table 1 and Table 2) [13,18-38]. These studies report median survival times between nine and 39 months.

The first neoadjuvant studies for patients with potentially resectable pancreatic cancer were performed in the early 1990s. In 1992, Evans *et al.* demonstrated the feasibility of radiation therapy combined with 5FU administration [35]. All patients were able to complete the neoadjuvant therapy, but grade 3 toxicities of 30% were reported. However, with this treatment, a resection rate of 61% was achieved. The median survival time of the patients whose tumors were resected was 18 months. These findings did not differ significantly from the results of adjuvant trials.

The application of other agents that are not usually used for the treatment of pancreatic cancer has been tested in some trials. For example, in a study conducted by Pisters *et al.* [25], the patients were treated with paclitaxel-based radiation given with a hypofractionated radiation regimen of 30 Gy in 10 fractions. They demonstrated the feasibility of the applied therapy, with a 46% incidence of grade 3 toxicity. Of the 35 treated patients, 20 underwent surgery and had their tumors resected (57%). The median survival time of the patients whose tumors were resected was 19 months. Compared with survival time of patients whose tumors were not resected, *i.e.*, 10 months, the result was not very satisfying.

Hoffmann *et al.* [21] initiated a neoadjuvant trial using a combination of 5FU and MMC (mitomycin C) in a cohort of 53 patients. These two agents were given concurrently with 50.4 Gy of radiation therapy. After finishing the protocol, 24 patients were able to undergo resection. The median survival time of the subjects who underwent surgery was 15.7 months, compared to 9.7 months for the whole group. Because of this regimen, 43% of patients developed grade 3-5 liver toxicity.

Numerous studies have examined the effect of combined 5FU plus cisplatin therapy in the neoadjuvant setting. Mourtardier *et al.* [30] enrolled 61 patients and treated them with a continuous infusion of 5FU and a bolus application of cisplatin. This regimen was combined concurrently with radiation therapy. Finally, only the tumors of 40 patients (66%) could be resected even though the therapy was completed by all enrolled patients. Therefore, it was a well-tolerated regimen without any grade 3–4 toxicity. The median survival time of the patients whose tumors were resected was 27 months, and the median survival time of the patients whose tumors were not resected was nine months. Thus, this study showed an advantage for preoperative treatment and demonstrated the feasibility of this treatment regimen.

Later, after many studies demonstrated the radiosensitizing effect of gemcitabine, numerous new studies were initiated. Wolff *et al.* treated 86 patients with 400 mg/m^2^ gemcitabine and a radiation dose of 30 Gy weekly. Of these patients, 61 underwent tumor resection (73%). Such high resection rates were only in a view other studies reported. Surprisingly, a median survival time of 36 months in the resected group was observed. In comparison to treatment with 5FU, the use of the combined therapy with gemcitabine showed a superior median survival time.

A similar resectability was found in a study conducted by Talamonti *et al.* [32]. The treatment was based on a full dose gemcitabine (1000 mg/m^2^) that was administered in combination with limited-field radiation at 36 Gy (2.4 Gy/fraction). After completion of the neoadjuvant therapy, 17 patients underwent surgery and had their tumors resected. Thus, a resection rate of 85% was reported. The median survival time of the resected subjects was 26 months. In comparison to early trials using 5FU, the excellent radiosensitizing effect of gemcitabine was demonstrated.

In a recent neoadjuvant trial performed by Evans *et al.*, preoperative radiotherapy was given to 86 patients at a dose of 30 Gy in 10 fractions over two weeks combined with the application of gemcitabine at doses of 400 mg/m^2^ seven times weekly [33]. After treatment, 73 patients (86%) underwent surgery, and 13 patients showed either progressive disease or an aggravation of the performance status. At the time of surgery, nine patients were found to have metastatic disease, and 64 patients (74%) underwent radical surgery. The median survival times and 5-year overall survival rates of all attended patients, resected patients and unresectable patients were 22.7 months and 27%, 34 months and 36%, and seven months and 0%, respectively.

Varadhachary *et al.* performed a further study involving 90 patients similar in concept to the study of Evans *et al.* In Varadhachary's study, induction chemotherapy was based on four cycles of cisplatin and gemcitabine following a 2-weekly schedule [34]. A respectable 88% of patients completed the whole treatment, and 66% underwent surgery and had their tumors resected. However, the median survival time, 17.4 months, was not improved. Although the median survival time of the patients whose tumors had been resected was 31 months, the addition of systemic therapy with gemcitabine and cisplatin did not result in a further improvement in survival.

The California Cancer Surveillance Program for Los Angeles County retrospectively considered 458 patients with resectable pancreatic adenocarcinoma who underwent definitive pancreatic resection and received systemic chemotherapy between 1987 and 2006. Using the data for these patients, the neoadjuvant and the adjuvant setting were compared. Of the 458 patients, 39 (8.5%) received neoadjuvant therapy, and 419 (91.5%) received adjuvant therapy. In the neoadjuvant treated group, a significantly better overall survival compared with the adjuvant group was observed (median survival time, 34 *vs.* 19 months, p = 0.003). In addition, a significantly lower rate of lymph node positivity was found in the neoadjuvant group (45% *vs.* 65%; p = 0.011). This analysis showed a clear benefit for the neoadjuvant-treated patients [39].

## 4. Neoadjuvant Therapy for the Treatment of Locally Advanced Disease

The definition of locally advanced disease is unresectability and the absence of distant metastasis. A uniform classification system for borderline or unresectable tumors has not been established.

Some studies have demonstrated the potential of neoadjuvant therapy to downstage patients with borderline resectable or unresectable pancreatic cancer. Table 2 lists a summary of the neoadjuvant trials for locally advanced pancreatic cancer [40-51].

In one of the first studies, Jessup *et al.* [40] reported the results of 16 patients who were treated with 5FU and 45 Gy radiotherapy. Disappointingly, only 12.5% (two patients) underwent surgery. However, these patients were disease free 20 and 22.5 months later.

In a retrospectively study conducted by Snady *et al.* [52], a group of patients who were treated with neoadjuvant therapy was compared to a group who underwent surgery alone. In the neoadjuvant setting, the patients received 5FU/streptozotocin/cisplatin and External Beam Radiation Therapy (EBRT) with 54 Gy followed by selective surgical resection (n = 68). The results of this study showed an advantage for patients receiving neoadjuvant therapy and subsequent surgery. Finally, 20 patients (29%) in the neoadjuvant group underwent resection. The median survival time was 14 months for the primary surgery group and was approximately 24 months in the neoadjuvant group. Therefore, the investigators demonstrated the feasibility of the regimen and an improvement of survival. One of the main criticisms of this study is that there was no objective, uniform classification for resectable or locally advanced disease.

Ammori *et al* [51] conducted a study that enrolled 67 patients with borderline and locally advanced tumors. These patients received neoadjuvant therapy based on chemotherapy with gemcitabine in combination with radiation therapy. Initially, of the 67 patients, 18 were classified as borderline resectable and 49 as unresectable. After completion of the preoperative therapy, patients underwent restaging. A total of 17 patients were restaged as resectable, 11 from the borderline resectable group and six from the group initially classified as unresectable. Ultimately, nine of these 17 patients (52%) underwent surgical resection; these were the only patients of the 67 to undergo resection. The median survival time of the patients whose tumors had been resected was 17.6 months, and the survival time of the remaining 58 unresected patients was 11.9 months. This study showed at least a small positive effect of using neoadjuvant therapy.

Pipas *et al.* treated 24 patients with a locally advanced stage pancreatic cancer with docetaxel and gemcitabine followed by gemcitabine-based chemoradiation [50]. Following completion of the neoadjuvant therapy, 17 patients underwent surgery and had their tumors resected. Only 13 of these patients had margin-negative resections.

Thus, neoadjuvant therapy can allow some patients with unresectable or borderline resectable disease to achieve resection with negative pathologic margins. The median survival has not been shown to improve significantly.

In a meta-analysis that compared 111 studies in the period from 1980 to 2009 retrospectively and prospectively, no significant difference with respect to the overall survival was found [53]. Considered were neoadjuvant radiochemotherapy, radiotherapy, or chemotherapy, followed by re-staging, and surgical exploration or resection. The group with resectable findings following treated with resection, showed similar survival rates compared to the resected patients who received adjuvant therapy. So no clearly advantage referring to neoadjuvant therapy was found. However, it was shown that one third of patients with initial locally advanced findings had an analogical survival rate after neoadjuvant therapy and resection in comparison to patients with initial resectable tumors [53].

Certainly, only randomized controlled trials can provide any level of evidence and can prove potential advantages or disadvantages.

## 5. Randomized Neoadjuvant Studies

The first multicenter randomized trial for neoadjuvant therapy for treatment of pancreatic carcinoma is currently recruiting patients [54]. This study will compare neoadjuvant gemcitabine/cisplatin-based chemoradiation plus resection with treatment with immediate resection in individuals whose disease is considered to be resectable at diagnosis. The resection will be followed by adjuvant chemotherapy in both arms. Because of the unique position of this study in the neoadjuvant setting, this study is highly relevant, and the results are eagerly awaited.

It is not clear why no other phase III randomized trials for neoadjuvant therapy for the treatment of pancreatic cancer have been initiated. Perhaps the recruitment of a sufficient number of patients is difficult.

## 6. Future Directions

The investigation of novel agents, for example, targeted therapies, such as Epidermal Growth Factor Receptor (EGFR) inhibitors, and anti-angiogenesis agents in combination with chemotherapy and/or radiation therapy, remains of great interest. In a single phase I study, the EGFR inhibitor gefitinib was used in combination with capecitabine and radiation therapy [55]. Additionally, another EGFR inhibitor, cetuximab, was applied in combination with gemcitabine [56].

The use of anti-VEGF monoclonal antibodies has been investigated in some trials. Kindler *et al.* demonstrated the feasibility of combining gemcitabine with bevacizumab. This study enrolled 52 patients with untreated advanced pancreatic cancer, of whom 11 (21%) had a partial response and 24 (46%) stable disease. The median survival time was 8.8 months, and no survival benefit was shown [57].

In a multicenter, randomized phase II trial, 84 eligible patients were randomly assigned to one of two treatment groups: weekly treatment with gemcitabine, cisplatin and cetuximab or weekly treatment with gemcitabine and cisplatin alone. There was no significant difference with respect to the survival rate between the two groups (7.8 months in the no cetuximab arm *vs.* 7.5 months in the cetuximab arm, p = 0.739) [58].

In addition, sorafenib, which inhibits VEGFR2 and Raf-1, was tested in a phase II trial in combination with gemcitabine. It was shown that sorafenib is well tolerated, but a median survival time of only four months was achieved [59].

Because of the disappointing results, it is necessary to find and test new therapeutic agents [60].

At the ASCO Annual Meeting in June 2010 a new randomized trial which compared gemcitabine to oxaliplatin and irinotecan plus fluorouracil and leucovorin (FOLFIRINOX) in patients with advanced pancreatic cancer was presented. This study showed significant improvements referring to progression free survival and median overall survival with FOLFIRINOX (6.4 months *vs.* 3.3 months and 11.1 months *vs.* 6.8 months, respectively) [61]. Furthermore an increased response rate (31 % for the FOLFIRINOX arm) in comparison to the gemcitabine arm (9 %) was pointed out. However this regime is more aggressive and has more side effects. Because of the promising results a neoadjuvant approach may be interesting.

## 7. Conclusions

Currently, the best chance to cure pancreatic cancer is the complete resection of the tumor. However, the achieved cure rates are not sufficient because most patients have advanced disease. Thus, neoadjuvant therapy for pancreatic cancer remains a theoretically attractive treatment strategy. Studies have demonstrated that patients can have a partial or complete response to neoadjuvant chemoradiation. Therefore, it is possible to reduce the incidence of margin positivity and to improve local control in selected patients. In conclusion, a survival benefit will be achieved.

The above-mentioned facts show the great potential of neoadjuvant therapy. The implementation of neoadjuvant studies is necessary to compare the different therapeutic strategies. However, the bureaucratic hurdles, especially in Germany, make it difficult to initiate new and innovative studies. Thus, it is extremely difficult to quickly move forward in this area. Ultimately, multicenter prospective randomized trials comparing the adjuvant and neoadjuvant approaches must be performed to determine the ideal treatment strategy for pancreatic cancer patients.

Although neoadjuvant therapy is not a disappointing approach, there is no satisfactory treatment at this time. Researchers must think ahead and continue to develop new and innovative issues.

## Figures and Tables

**Table 1.** Neoadjuvant Therapy for Resectable Disease.

**Series**	**Year**	**Number**	**Regimen**	**Resection Rate**		**m OS (Months)/all**	**m OS (Months)/res**	**m OS (Months)/n-res**	**p-value**
				**N**	**%**				
Evans *et al.*	1992	28	EBRT + 5FU (± IORT)	17/28	61	-	18	-	-
Yeung *et al.*	1993	26	EBRT + 5FU + MMC	12/26	46	8	12	-	-
Ishikawa *et al.*	1994	23	EBRT	17/23	74	15	15	9	-
Staley *et al.*	1996	39	EBRT + 5FU (± IORT)	33/39	85	19	-	-	-
Spitz *et al.*	1997	91	EBRT + 5FU	52/91	57	19.2	-	-	-
Pisters *et al.*	1998	35	EBRT + 5FU + IORT	20/35	57	-	25	7	-
Hoffmann *et al.*	1998	53	EBRT + 5FU + MMC	24/53	45	9.7	16	-	-
White *et al.*	2001	53	EBRT + 5FU + MMC + CP	28/53	53	22	-	-	-
Breslin *et al.*	2001	132	EBRT + 5FU, or Pac or Gem	-	-	21	-	-	-
Wolff *et al.*	2002	86	EBRT + Gem	63/86	73	-	36	-	-
Moutardier *et al.*	2002	19	EBRT + 5FU + CP	15/19	79	20	30	-	-
Pisters *et al.*	2002	35	EBRT + Pac + IORT	20/35	57	12	19	10	-
Magnin *et al.*	2003	32	EBRT + 5FU + CP	19/32	59	16	30	-	-
Sasson *et al.*	2003	116	EBRT + 5FU/MMC or Gem	-	-	18	-	-	-
		*61 neo*				*23*			
		*55 adj*				*16*			
Calvo *et al.*	2004	15	EBRT + Tegafur + IORT	9/15	60	17	23	8	0.02
Moutardier *et al.*	2004	61	EBRT + 5FU + CP	40/61	66	23	27	9	-
Meszoely *et al.*	2004	63	EBRT + Gem	41/63	65		20		
White *et al.*	2005	193	EBRT + 5FU or Gem	70/193	36	23	-	-	-
		*102 ir*		*54/102*	*53*	*39*			
		*91 ilu*		*16/91*	*18*	*20*			
Mornex *et al.*	2006	41	EBRT + 5FU + CP	26/41	63	9,4	11.7	8.5	-
Talamonti *et al.*	2006	20	EBRT + Gem	17/20	85	-	26	-	-
Vento *et al.*	2007	29	EBRT + Gem			-			
		*15 neo*		*13/15*	*87*	*-*	*27*		
		*14 Op*				*-*	*20*		
Evans *et al.*	2008	86	EBRT + Gem	64/86	74	22.7	34	7.1	<0.001
Varadhachary *et al.*	2008	90	CP + Gem + EBRT + Gem	52/79	66	17.4	31	10.5	<0.001
Turrini *et al.*	2009	101	EBRT + 5FU + CP	62/101	61	17	23	11	0.002

EBRT = External Beam Radiation Therapy; IORT = Intraoperative Radiation Therapy; 5FU = 5-Fluorouracil; CP = Cisplatin; Gem = Gemcitabine; MMC = Mitomycin C; STZ = Streptozotocin; Pac = Paclitaxel; m OS = median overall survival; res = resected; n-res = non-resected

**Table 2.** Neoadjuvant Therapy for locally advanced Disease.

**Series**	**Year**	**Number**	**Regimen**	**Resection Rate**		**m OS (Months)/all**	**m OS (Months)/res**	**m OS (Months)/n-res**	**p-value**
Jessup *et al.*	1993	16	EBRT + 5FU	2/16	12.5	8	-	-	-
Kamthan *et al.*	1997	35	EBRT + 5FU + STZ + CP	5/35	14	15	31	11	-
Todd *et al.*	1998	38	5FU + MMC + Dipyridamole	4/38	10.5	15.5	-	-	-
White *et al.*	1999	25	EBRT + 5FU + MMC + CP	5/25	20	12	-	-	-
Wanebo *et al.*	2000	14	EBRT + 5FU + CP	9/14	64	-	16	9	-
Kastl *et al.*	2000	27	EBRT + 5FU + MMC	10/27	37	11	-	-	-
Snady *et al.*	2000	159	EBRT + 5FU + STZ + CP						0.006
		68 neo		20/68	29	23.6	32.3	21.2	-
		91 only Op				14	-	-	-
Ammori *et al.*	2003	67	EBRT + Gem ± CP	9/67	13	-	17,6	11.9	-
Aristu *et al.*	2003	47	EBRT + 5FU ± Pac; CP + 5FU; Docetaxel + Gem	9/47	19	11	23	10	-
Joensuu *et al.*	2004	28	EBRT + Gem	20/28	71	25	25	14	-
White *et al.*	2004	88	EBRT + 5FU + CP + MMC	18/88	20	-	23	-	-
Pipas *et al.*	2005	24	EBRT + Gem + Doc	17/24	70	14	-	-	-
Tinkl *et al.*	2008	120	EBRT + 5FU + MMC or EBRT + Gem + CP	38/120	32	25	-	-	-

EBRT = External Beam Radiation Therapy; 5FU = 5-Fluorouracil; CP = Cisplatin; Gem = Gemcitabine; MMC = Mitomycin C; STZ = Streptozotocin; Pac = Paclitaxel; Doc = Docetaxel; m OS = median overall survival; res = resected; n-res = non-resected
